# Role of StdA in adhesion of *Salmonella enterica* serovar Enteritidis phage type 8 to host intestinal epithelial cells

**DOI:** 10.1186/1757-4749-5-43

**Published:** 2013-12-24

**Authors:** Daniel C Shippy, Nicholas M Eakley, Dareen M Mikheil, Amin A Fadl

**Affiliations:** 1 Department of Animal Sciences, University of Wisconsin-Madison, 1675 Observatory Drive, Madison, WI 53706, USA

**Keywords:** *Salmonella*, Adhesion, StdA, Poultry

## Abstract

**Background:**

*Salmonella* is often implicated in foodborne outbreaks, and is a major public health concern in the United States and throughout the world. *Salmonella enterica* serovar Enteritidis (SE) infection in humans is often associated with the consumption of contaminated poultry products. Adhesion to epithelial cells in the intestinal mucosa is a major pathogenic mechanism of *Salmonella* in poultry. Transposon mutagenesis identified *stdA* as a potential adhesion mutant of SE. Therefore, we hypothesize StdA plays a significant role in adhesion of SE to the intestinal mucosa of poultry.

**Methods and results:**

To test our hypothesis, we created a mutant of SE in which *stdA* was deleted. Growth and motility were assayed along with the *in vitro* and *in vivo* adhesion ability of the ∆*stdA* when compared to the wild-type SE strain. Our data showed a significant decrease in motility in ∆*stdA* when compared to the wild-type and complemented strain. A decrease in adhesion to intestinal epithelial cells as well as in the small intestine and cecum of poultry was observed in ∆*stdA*. Furthermore, the lack of adhesion correlated to a defect in invasion as shown by a cell culture model using intestinal epithelial cells and bacterial recovery from the livers and spleens of chickens.

**Conclusions:**

These studies suggest StdA is a major contributor to the adhesion of *Salmonella* to the intestinal mucosa of poultry.

## Background

*Salmonella* is a significant foodborne bacterium associated with enteric disease outbreaks in humans due to the consumption of contaminated food. *Salmonella* serovars, like *Salmonella enterica* serovar Enteritidis (SE), are the leading cause of death among the major foodborne pathogens [1]. SE phage type (PT) 8 is one of the most common PTs associated with egg-associated outbreaks in the United States while SE PT4 is the most common in Europe [2,3]. Therefore, the identification and evaluation of *Salmonella* virulence factors could help develop new ways to control salmonellosis in the farm to fork food processing cycle.

A hallmark of *Salmonella* virulence is its ability to invade host intestinal epithelial cells [4]. This is a multi-step process mediated by a type 3 secretion system (T3SS) encoded within *Salmonella* pathogenicity island-1 (SPI-1) [5,6]. The first step in the invasion process is the adhesion of *Salmonella* to the host intestinal epithelial cells. Several pathogenic factors have been implicated in adhesion to host cells. The best characterized are the fimbrial adhesins which include type 1, plasmid-encoded, long polar, and thin aggregative fimbriae [7-10]. A further study has suggested that the T3SS itself can mediate host cell adhesion by showing that SipB, SipC, and SipD are required for the intimate association of *Salmonella* with mammalian cells [11]. Inhibition of *Salmonella* adhesion at the initial stages of infection is potentially the most effective strategy for controlling salmonellosis in production animals which could result in reduced contamination of our food supply [12].

In this study, we identified *stdA* as an adhesion mutant of SE by transposon mutagenesis. The *stdA* deletion mutant (∆*stdA*) displayed a normal growth profile when compared to the wild-type (WT) SE PT8 and complemented strains. A motility assay showed a significant decrease in motility for ∆*stdA.* Adhesion and invasion assays showed ∆*stdA* was deficient in cell culture models of *Salmonella* adhesion and invasion. Furthermore, ∆*stdA* was deficient in a poultry model of *Salmonella* adhesion and invasion with the systemic infection deficiency most likely due to the decreased adhesion. Taken together, these data indicated a major role for StdA in the adhesion ability of SE host cells.

## Results

### Analysis of ∆*stdA*

The chromosomal *stdA* gene was replaced by a kanamycin resistance gene (Kn^R^) cassette using the lambda Red recombination system. Deletion of *stdA* from the chromosome of SE was confirmed by PCR analysis. The primer set K_3_/K_5_ was used to amplify the Kn^R^ cassette, while the primer set F_2_/R_2_ was used to confirm the absence of *stdA*. To ensure correct orientation of the Kn^R^ cassette, the primer set F_3_/K_5_ was used to amplify the upstream *stdA* flanking sequence along with the Kn^R^ cassette, while R_3_/K_3_ was used to amplify the downstream *stdA* flanking sequence along with the Kn^R^ cassette. Overall, these results indicated that a *stdA* deletion mutant of SE PT8 was successfully created.

### StdA does not affect *Salmonella* growth

**Figure 1:**
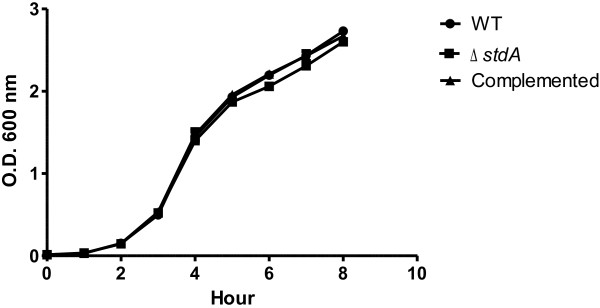
**Growth curves of the WT, ∆*stdA*, and complemented strains.** The strains were grown in LB and the optical densities at 600 nm were measured each hour. The graph is representative of two independent experiments.

Growth curve analysis was conducted for the WT, ∆*stdA,* and complemented strains in order to determine the relevance of StdA on *Salmonella* growth. All three strains displayed nearly identical growth profiles suggesting StdA does not play a significant role in SE growth (Figure 1).

### ∆*stdA* is deficient in motility

**Figure 2:**
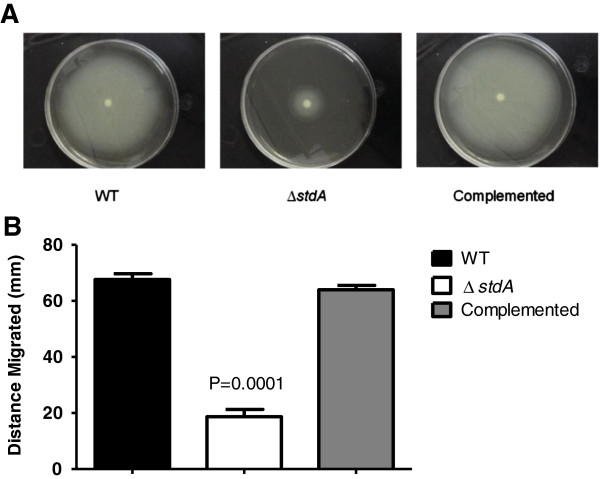
**Motility assay of the WT, ∆*stdA*, and complemented strains.** Bacteria were spotted onto soft agar, and migration of the bacteria was measured from the inoculation point to the periphery of the plate. **(A)** Images showing the migration of each SE strain. **(B)** Graph displaying the migration of each SE strain. The actual *P* values are given displaying a statistically significant difference between ∆*stdA* and the WT strain.

A motility assay was conducted to see if StdA has a role in SE motility. Measurement of the motility plates displayed a significantly reduced migration from the inoculation site to the periphery of the plate for ∆*stdA* (19 mm) when compared to the WT (68 mm) and complemented (64 mm) strains (Figure 2).

### ∆*stdA* is attenuated in adhesion and invasion *in vitro*

**Figure 3:**
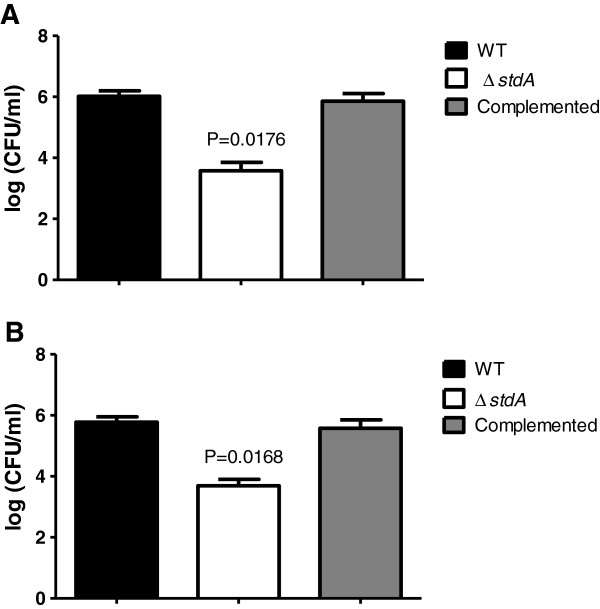
**Cell culture assays using T84 intestinal epithelial cells. (A)** Adhesion assay and **(B)** invasion assay with the WT, ∆*stdA*, and complemented strains. The actual *P* values are given displaying a statistically significant difference between ∆*stdA* and the WT strain.

Inoculation of intestinal epithelial cells displayed a significant decrease in adhesion ability in ∆*stdA* (3.6 logs) when compared to the WT (6.0 logs) and complemented strains (5.9 logs) (Figure 3A). Furthermore, ∆*stdA* displayed the same significant decrease in the ability to invade T84 intestinal epithelial cells (Figure 3B). The adhesion and invasive ability of ∆*stdA* was restored after complementation, suggesting StdA plays a role in adhesion and invasion of intestinal epithelial cells by SE. It is logical to conclude that the invasion defect seen in ∆*stdA* is an effect of the adhesion deficiency.

### Deletion of *stdA* attenuates SE adhesion and invasion in chickens

**Figure 4:**
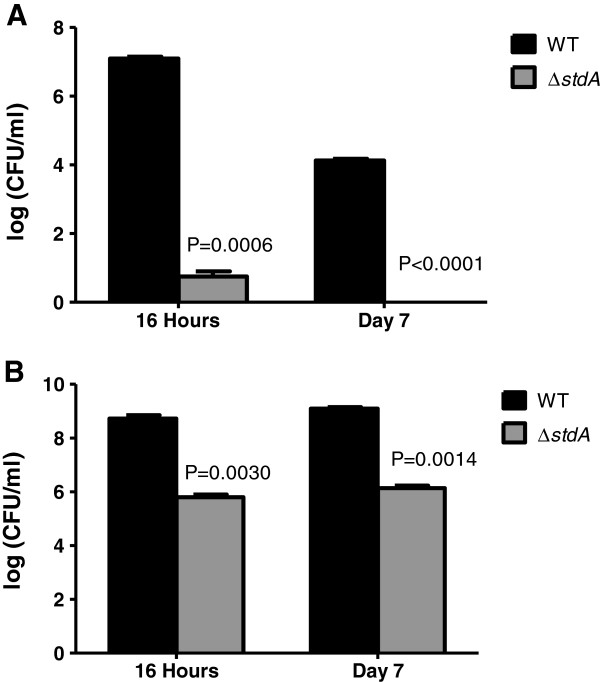
**Determination of adhesion ability in poultry.** Bacterial counts in **(A)** small intestine and **(B)** cecum of chickens inoculated by oral crop gavage with 1 × 10^7^ CFU of WT and ∆*stdA*. The actual *P* values are given displaying a statistically significant difference between ∆*stdA* and the WT strain.

A chicken model of infection was used to determine the role of StdA in the adhesion ability of SE to the intestinal mucosa of chickens. At 16 hours post-infection, the bacterial counts of WT SE from the small intestine were 7.1 logs compared to 0.75 logs for ∆*stdA.* At day 7 post-infection, bacterial counts from the small intestine were 4.1 logs for the WT with no bacteria recovered for ∆*stdA* (Figure 4A). For the cecum, bacterial counts from chickens infected with the WT SE strain were 8.7 and 9.1 logs at the 16 hour and day 7 time points, respectively compared to 5.8 and 6.1 logs for ∆*stdA* (Figure 4B)*.* These data suggest StdA plays a role in the adhesion ability of SE to the intestinal mucosa of chickens.

**Figure 5:**
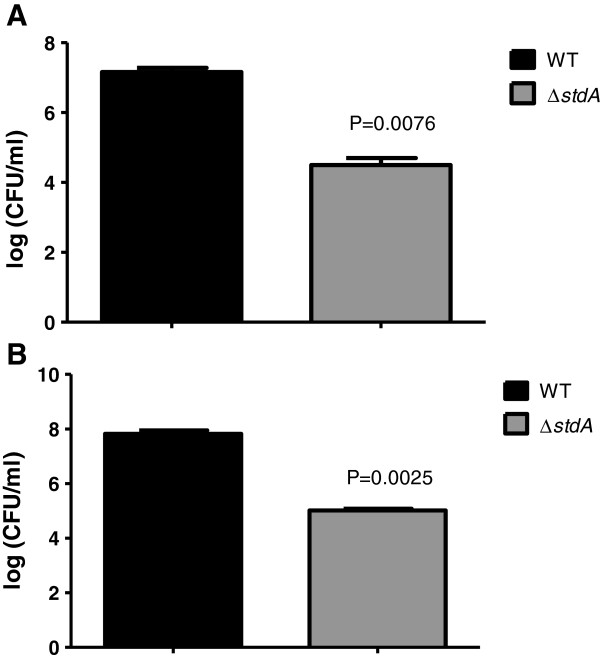
**Systemic infection ability of the WT and ∆*stdA *SE strains.** Bacterial counts in **(A)** liver and **(B)** spleen of chickens inoculated by oral crop gavage with 1 × 10^7^ CFU of WT and ∆*stdA*. There was no bacterial recovery at the 16 hour time point from either the liver or spleen of chickens inoculated with the WT or ∆*stdA* strain, so only the day 7 data is shown. The actual *P* values are given displaying a statistically significant difference between ∆*stdA* and the WT strain.

We also assayed the bacterial counts from the livers and spleens to see if the adhesion deficiency displayed by ∆*stdA* affected systemic infection in chickens. At the 16 hour time point, there was no bacteria recovered from the livers and spleens of chickens infected with the WT and ∆*stdA* strains. At the day 7 time point, bacterial counts in the liver were 7.2 and 4.5 logs comparing the WT and ∆*stdA,* respectively (Figure 5A). Furthermore, bacterial counts in the spleen were 7.8 and 5.0 logs comparing the WT and ∆*stdA,* respectively (Figure 5B). These data suggest that the adhesion deficiency displayed by ∆*stdA* contributes to an overall reduction in systemic infection by SE in poultry.

## Discussion

In this study, an adhesion mutant of SE was created and characterized. Transposon mutagenesis identified StdA as a potential adhesion mutant of SE. A ∆*stdA* strain of SE was created using the lambda Red recombination system, and was deficient in adhesion in both cell culture and chicken models of infection. Additionally, this adhesion defect lead to a deficiency in invasion of T84 intestinal epithelial cells, and decreased overall systemic infection ability in a poultry model as evidenced by reduced bacterial counts in the livers and spleens of chickens inoculated with ∆*stdA*. These data indicated that StdA plays a significant role in the adhesion ability of SE to the intestinal mucosa of poultry.

StdA is a 19-kDa fimbrial protein that is part of the *std* operon which was originally identified during sequence analysis of the *Salmonella enterica* serovar Typhi CT18 strain [13]. It was later found to be in other serovars of *Salmonella* including *Salmonella enterica* serovar Typhimurium (STM) [14-17]. In STM, the Std fimbriae play a role in *Salmonella* adhesion to specific sections of the intestinal mucosa as evidenced by *std* operon deletion mutants having reduced intestinal persistence in mice [18,19]. This correlates to the data observed in our study, where deletion of *stdA* significantly alerted the adhesion ability of SE in the intestinal mucosa of poultry.

The synthesis of Std fimbriae is tightly regulated, but the mechanisms involved in *std* expression are unclear. In the study by Balbontin *et al.,* gene expression profiling of a *dam* mutant of STM demonstrated that transcription of the *std* operon is repressed by Dam methylation [20]. In another study, Jakomin *et al.* showed that uncontrolled expression of Std fimbriae contributes to the attenuated virulence observed in *dam* mutants of STM [21]. They also described a regulatory role for SeqA as a repressor of the *std* operon and HdfR as an activator of *std* expression whose activity may be antagonized by SeqA [21]. Further regulatory evidence was displayed in the study by Chessa *et al.* which identified RosE as a novel transcriptional regulator of Std fimbrial expression in STM [18]. Further investigation into the regulation of *stdA,* and how it affects *Salmonella* adhesion to the intestinal mucosa of poultry will be conducted in our laboratory.

An interesting observation in our study is that ∆*stdA* displayed a significant decrease in motility. Motility is hypothesized to be a pathogenic mechanism because it promotes contact with the surface of epithelial cells by allowing the bacterium to penetrate the thick mucus layer covering the intestinal mucosa [22,23]. Some studies suggest a role for flagella in bacterial adhesion to host tissue [24,25]. The study by Erdem *et al.* suggests a role for FliC in *E. coli* adhesion to bovine intestinal tissue while the study by Olsen *et al.* suggests a role for FliC in *Salmonella* binding to intestinal epithelial cells [24,25]. Further studies will be needed to determine how StdA affects *Salmonella* motility and if this motility reduction contributes to the adhesion and invasion defect seen in ∆*stdA.*

Additional studies will also be needed in order to gauge the level of attenuation of the ∆*stdA* SE strain in chickens. Depending on the outcome of these studies, further studies could be conducted to determine if ∆*stdA* is a good candidate for use in a live-attenuated poultry vaccine.

## Conclusions

Transposon mutagenesis identified StdA as a potential adhesion mutant of SE. A ∆*stdA* strain of SE was created using the lambda Red recombination system, and was deficient in adhesion both *in vitro* and *in vivo*. Additionally, this lack of adhesion lead to a deficiency in invasion of T84 intestinal epithelial cells, and decreased overall systemic infection ability in a poultry model as evidenced by reduced bacterial counts in the livers and spleens of chickens inoculated with ∆*stdA*. Overall, our data suggest StdA plays a role in the adhesion ability of *Salmonella* to the intestinal mucosa of chickens, and could be an important factor in the early stages of *Salmonella* infection in poultry.

## Methods

### Bacterial strains, plasmids, and cell lines

The WT SE PT8 E2627 strain was isolated from an egg-associated outbreak in the United States [26]. All *Salmonella* strains were grown in either Luria-Bertani (LB) medium or on *Salmonella-Shigella* (SS) plates. Additionally, all homogenates from the *in vivo* experiments were incubated in Selenite-F broth (BD, Sparks, MD). Nalidixic acid (100 μg/ml), kanamycin (50 μg/ml), tetracycline (15 μg/ml), and ampicillin (100 μg/ml) were added to the media as necessary. A complete list of the bacterial strains and plasmids used in this study is shown in Table 1. T84 intestinal epithelial cells were obtained from the American Type Culture Collection (ATCC, Manassas, VA), and were subsequently grown and maintained in Dulbecco’s Modified Eagle Medium: Nutrient Mixture F12 (DMEM/F12) medium supplemented with 10% fetal bovine serum (FBS), and incubated at 37°C with 5% CO_2_.

**Table 1:** Strains and plasmids used in this study

**Strain or plasmid**	**Relevant characteristics**	**Source or reference**
Serovar Enteritidis phage type 8 E2627	Isolated from an egg-associated outbreak in the United States	[26]
∆*stdA*	Mutant of serovar Enteritidis in which *stdA* was deleted using lambda Red; Kn^r^	This study
∆*stdA/*pBR*stdA*	∆*stdA* complemented with a copy of the *stdA* gene via pBR322; Kn^r^, Tc^r^	This study
***E. coli***		
DH5α	Used for recombinant DNA methods	Lab stock
**Plasmids**		
pKD46	lambda Red recombinase genes; Ap^r^	Lab stock
pBR322	Ap^r^ Tc^r^	Lab stock
pKD4	Kn^r^ gene cassette	Lab stock
pBR*stdA*	*stdA* gene cloned into pBR322 at the *ScaI* site	This study

### Construction of the mutant and complemented strains

The transposon binding screening which identified ∆*stdA* as a potential binding mutant of SE is described in [27]. The ∆*stdA* strain was created using the lambda Red recombination system as previously described [28]. Briefly, WT SE PT8 was transformed with the pKD46 plasmid that carries the lambda Red recombinase genes [28]. Arabinose-induced WT SE carrying pKD46 was cultured and used to generate the electrocompetent cells. The kanamycin resistance gene (Kn^R^) was PCR amplified from the pKD4 plasmid using primer set LF/LR [28]. The 5′-end of the LF primer carries 40 extra bases homologous to the upstream *Salmonella stdA* gene while the 5′-end of the LR primer carries 40 bases homologous to the downstream *stdA* flanking sequence. The PCR product was purified and electroporated into the WT-pKD46 electrocompetent cells. After transformation, colonies growing on LB plates supplemented with kanamycin were selected as candidates for *stdA* mutants of SE. To confirm deletion of the *stdA* gene, the selected mutants were subjected to PCR analysis using primer sets K_3_/K_5_ and F_2_/R_2_ to show the presence of the Kn^R^ and the absence of *stdA.*

The *stdA* complemented strain was constructed by amplifying a DNA fragment containing *stdA* from the WT SE strain using primer set F_3_/R_3_. The DNA fragment was blunt-ended using a PCR polishing kit (Stratagene, Santa Clara, CA) and ligated into the blunt-ended Sca*I* restriction enzyme digested pBR322 vector. The recombinant plasmid was transformed into the *stdA* mutant by electroporation. A complete list of the primers used in this study is shown in Table 2.

**Table 2:** Sequence and purpose of primers used in this study

**Primer name and sequence**	**Purpose**
LF:5′-AAAGGACATATTATCTATGCGTAATAAAATAATACTTGCCTGTGTAGGCTGGAGCTGCTT-3′	Forward primer for amplification of the Kn^R^ gene cassette and upstream *stdA* flanking sequence
LR:5′-CCGTGGACGGCTTCTCCCTGTCGTTATTTACCGCGTGAAACATATGAATATCCTCCTTAG-3′	Reverse primer for amplification of the Kn^R^ gene cassette and downstream *stdA* flanking sequence
F_2_:5′-CATCACCAACTCACCCTGTG-3′	Forward primer for amplification of the *stdA* gene
R_2_:5′-CTGAGGTATCTGCTGTGCCA-3′	Reverse primer for amplification of the *stdA* gene
F_3_:5′-ATTCATATGGTGCTTCGTTTAACACC-3′	Forward primer for amplification of *stdA* for complementation
R_3_:5′-AGACTCGAGTCACAGGTATTTCAGG-3′	Reverse primer for amplification of *stdA* for complementation
K_3_: 5′-AAAGCCACGTTGTGTCTA-3′	Forward primer for amplification of the Kn^r^ gene cassette
K_5_: 5′- CGCTGAGGTCTGCCTCGT-3′	Reverse primer for amplification of the Kn^r^ gene cassette

### Growth analysis

Growth curve profiles were constructed in order to determine the significance of StdA on SE growth. An equal number of cells from the WT, ∆*stdA,* and complemented strains were inoculated in LB and grown at 37°C. The optical densities at 600 nm were recorded each hour.

### Motility assay

The motility assay was performed as previously described [29]. Briefly, soft agar (LB medium with 0.3% agar) was used to characterize the motility phenotype of the WT, ∆*stdA*, and complemented SE strains. Overnight cultures of each *Salmonella* strain were adjusted to the same optical density. Equal numbers of CFU (1 × 10^6^) were spotted onto 0.3% LB agar. The plates were incubated at 37°C, and motility was determined by examining the migration of the bacteria from the center of the inoculation point to the periphery of the plate.

### Adhesion assay

The adhesion assay was performed as previously described [30]. Briefly, 5 × 10^5^ T84 intestinal epithelial cells were seeded per well in a 24-well tissue culture plate and incubated overnight at 37°C with 5% CO_2_. The following day, cells were infected with the WT, ∆*stdA,* and complemented strains at a multiplicity of infection (MOI) of 10:1. The plate was briefly centrifuged, and incubated for 30 minutes at 37°C with 5% CO_2_. Unbound bacteria were aspirated; the wells washed six times with phosphate buffered saline (PBS), and the cells were lysed with 0.1% Triton X-100 (TX-100). Dilutions of the cell lysates were plated on SS agar for enumeration of bacteria.

### Invasion assay

The invasion assay was performed as previously described [30]. Briefly, 5 × 10^5^ T84 intestinal epithelial cells were seeded per well in a 24-well plate and incubated overnight at 37°C with 5% CO_2_. The cells were infected with the WT, ∆*stdA,* and complemented strains at an MOI of 10:1, and briefly centrifuged so that the bacterial cells would be in direct contact with the T84 cells. After incubation for 30 minutes at 37°C with 5% CO_2_, the cells were washed three times with PBS and incubated for an additional 45 minutes with gentamicin-containing medium (100 μg/ml) to kill extracellular bacteria. Following incubation, the gentamicin-containing medium was removed, the wells were washed six times with PBS, and the cells were lysed with 0.1% TX-100. The lysate was diluted and plated out on SS agar plates for colony-forming unit (CFU) determination.

### Chicken experiments

One-week-old specific-pathogen-free (SPF) White Leghorn chickens were purchased from Charles River (Wilmington, MA). Groups of 11 birds were infected by oral crop gavage with 1 × 10^7^ CFU of the WT or ∆*stdA* SE strains. Another group of birds (n = 4) was inoculated by oral crop gavage with 100 μl sterile PBS to serve as a control. At 16 hours and 7 days post-infection, 5 birds from each group were euthanized using CO_2._ Portions of the liver, spleen, small intestine, and cecum were removed from each bird. The individual organs were pooled and 1 gram was homogenized in 10 ml PBS. One ml from each homogenate was incubated in 10 ml Selenite-F broth at 36°C for 18 hours. Direct plating of the organ homogenates was done in parallel with plating from the enrichment cultures [31-34]. Enumeration of bacteria was performed by serial dilution from the Selenite-F broth and plating on SS agar.

### Statistical analysis

Wherever appropriate, the data were analyzed using GraphPad Prism 5 software (GraphPad Software, San Diego, CA) and a Student’s *t* test. *P* values of ≤ 0.05 were considered significant. Unless otherwise stated, experiments were repeated two times and data were expressed as arithmetic means with standard deviations.

## Competing interests

The authors declare that they have no competing interests.

## Authors’ contributions

DS carried out the *in vivo* and *in vitro* experimental work, performed the statistical analysis, and drafted the manuscript. NE performed the transposon screening and identified the adhesion mutants. DM created the *stdA* mutant strain. AF designed and coordinated the study, and edited the manuscript. All authors read and approved the final manuscript.
